# Address of Judge Nisbet at the Commencement Exercises of the Southern Medical College, February 29, 1888

**Published:** 1888-04

**Authors:** 


### ADDRESS OF JUDGE NISBET AT THE COMMENCEMENT EXERCISES OF THE SOUTHERN MEDICAL COLLEGE, FEBRUARY 29, 1888.

Judge Nisbet was greeted with applause as he approached the front of the stage and placed the manuscript of his address upon the lecturn. He spoke as follows:

"In the mythological legends of Greece, Esculapius, the first distinguished devotee of the healing art, was the god of medicine. Magnificent temples were erected in his honor, with priests and priestesses, where costly votive offerings were made, and gorgeous ceremonies were presented with every adjunct of darkness and of mystery to impose upon the suffering and credulous worshipers. Even centuries after, when the eastern world had attained an excellence in the fine arts, in poetry, sculpture, architecture and everything indicative of a high æsthetic culture, which has never been reached in modern times, the practice of medicine continued to be a system of superstitious frauds and impositions, in comparison with which the methods of the street fakir are rational and honest. And it has only been in comparatively recent times that medicine has exhibited a progress equal to that made in other branches of human knowledge.

The medical profession was retarded for ages by the prejudice of pagan and Christian against dissection of the human body. Its progress has been slow, too, because of the uncertainty as to results, incident to the application of the most exact knowledge under conditions as shifting as the sands upon the sea-shore, and as various as the idiosyncracies of individuals, and the diseases to which flesh is heir. Besides there is so much that is occult in diseases and their remedies, and so much that is indeterminate and indeterminable in methods of treatment—so much ignorance of the subjects involved among the masses of the people, that the profession has always been, and is to-day, the favorite arena of all kinds of pretenders and impostors, whose ignorance can be measured only by their impudence. A vast multitude of these characters—charlatans, empyrics, specialists, vendors of patent nostrums, impostors of every kind, and of both sexes, from the woman who cures you by manipulation of your epidermis, to the man who cures you by faith—"the substance of things hoped for and the evidence of things not seen"—swarm along the path of the profession like the rabble which follows the march of a regular army. They outnumber the regular practitioners, as the barbaric hosts of their savage allies outnumbered the Roman legions in Gaul. They are the camp followers of the profession, hanging like a cloud upon its flanks and rear, but worse than the camp followers of an army, because they impede its progress, draw its rations and its pay, and whenever occasion offers, turn their arms against it

But, in the last one hundred years the advance in medical knowledge has been extraordinary, and its history is full of magnificent discoveries, useful inventions and startling revelations. Within that period diseases which were the scourges of the human race, on the land and upon the sea, have ceased to be formidable. Pain has been rendered painless by anæsthetic agents. Pharmaceutical chemistry has enriched the materia medica with a variety of new remedies, and furnished the profession with improved weapons with which to combat disease. New and improved methods, and new and improved instruments in investigating disease, have rendered diagnosis easier and more certain, and a series of useful inventions and startling discoveries, to which the profession in Georgia has made splendid contributions, has illustrated the history of medicine, and it is safe to predict that, in the next one hundred years, the progress of the medical profession will be equally brilliant, for there are few branches of human knowledge which can be extended without adding something to the progress of medical science.

Many of the difficulties by which the medical profession is beset might be materially lessened if it had a little recognition and protection from the government. The Federal Government has paid more money in the investigation of pleuro pneumonia and cholera in swine than for scientific research into all the diseases to which human beings are subject. In Georgia there is practical free trade in the manufacture, sale, administration and practice of medicine, and practically no protection is furnished by our laws either to practitioners or to the people. In this state, and under its code, health and life are made subordinate to property and a great many other things. There are upon the statute book elaborate laws for the inspection of illuminating oils, with severe penalties for their violation, but none for drugs and medicines, whose administration may re-illume the flickering light of life or put it out forever. There are statutes for the inspection of fertilizers and special officers to enforce them and severe penalties for their violation, but their are no laws for the analysis and inspection of the tons of "perilous stuff" which goes into the stomachs of the people when there stomachs are sick. No dissolved bone can be sold in Georgia unless it is shown by official analysis to contain at least ten per cent. of available phosphoric acid. No ammoniated super-phosphate can be sold in Georgia unless it is shown by official analysis to contain, at least, eight per cent. of available phosphoric acid and two per cent. of ammonia. But there is no law for the analysis or inspection of thousands of mortal drugs and leprous distilments, taken by the people, in sickness and in health, to ascertain whether they come up to any standard of purity or of strength. Manures in Georgia are of more importance than medicines. The earth is fed with more care and caution in Georgia than the men, women and children who live upon it. The cultivation of the soil in Georgia is of more importance than the health and happiness and lives of the people. Crops are cared for more tenderly than children. There are laws upon the statute book against the carrying of concealed weapons, but no law which forbids the manufacture and sale, and prescription of a thousand drugs and medicines, whose properties are concealed, but which are more destructive of human life than a whole battery of Gatling guns. There are laws upon the statute book against cruelty to animals, and they define "cruelty" to include "every wilful act, omission or neglect, whereby unjustifiable physical pain, suffering or death is caused or permitted." And yet our laws tolerate cruelty to men and women, and permit unjustifiable physical pain, suffering and death to be inflicted upon them, provided the crime is committed with a drug; until dying is as much a mystery as living, and living is a miracle. Georgia, I repeat, has practically free trade in the manufacture, sale and administration of medicine, and does not furnish one particle of protection to its practitioners or their patients. The law requiring a diploma to practice medicine is a dead letter, and under the definition given in the code of what is meant by practicing medicine, is violated every day with perfect impunity. One specialty may be practiced by women without a license. Georgia has no medical board to guard the entrance into the temple where Hygeia is worshipped, to protect it from profanation, and to protect the graduates of this and other medical institutions of high standing, and enable them to enjoy the full benefits of the time, the talent, the learning and the conscientious labor which has been expended in fitting them for their responsible duties. It has no Board of Health (it died of inanition several years ago) to preserve vital statistics and to take control of all the important matters connected with the public health. There can be no subjects of more vital interest to the people of Georgia—none more deserving the attention of legislators; and the practitioners and professors of medicine, and the pharmacists who are skilled in their business, and conscientious in its conduct, who together constitute so large and intelligent and influential a class of the people, can do no higher service to the state, to humanity, or to the profession of medicine, than to present them to the attention of the next general assembly. By an organized effort they may induce that body to devote to these important subjects, some of the time and attention which it usually gives so generously to matters connected with per diem and the next election.

As to the practitioners of this noble science, it is difficnlt to use any language of eulogy which would be extravagant. I do not refer to that horde of charlatans and impostors who hang about the outskirts of the profession, traffickers in human blood, and speculators upon the ignorance of the credulous and the fears of the timid, nor to those members of the profession whose professional studies cease with their graduation, nor to those who seem to be hardened by their professional studies and pursuits, and have "lost all feeling of their calling" like the grave digger in Hamlet, who sings as he digs and jests as he throws out a skull. These are not true representatives of the medical profession. As well might we select that class of members of the bar, satirized by Martial, as men who hire out their words and anger (iras et verba locant) as fair representatives of the noble profession of the law. I do not refer to them, but to that large class of earnest, able, conscientious, laborious, cultivated and studious gentlemen, who consecrate their lives, and their talents, and their energies to the duties of their profession.

The occupation in which they are engaged associates them constantly with the better part of creation, and subjects them constantly to the refining, ennobling and humanizing influence of woman. It presents every day opportunities for charity, and for the exercise of the best and noblest impulses of our nature. It cultivates constantly those principles and feelings and habits of gentleness, courtesy, sincerity and bravery, which constitute the true gentleman. It requires labors which tax the strongest constitution; reasonings and investigations which require the strongest intellect and the broadest culture; independence of thought and of action which demand a robust manliness; and the result among its worthy devotees is the development of a type of man and gentleman higher than is often found in any other secular occupation.

And this is true notwithstanding physicians, in the discharge of their duties, lack many of the incentives which influence other men to great and noble deeds. Their arena of action is circumscribed. The subjects upon which they act are usually of interest only to individuals. They move much along untrodden ways, far from the maddening crowd, and play their parts upon a narrow stage, where there are few spectators to applaud them. They have little more to inspire them or to strengthen their nerve than a sense of duty and a consciousness of right. In comparison, how complex and how powerful are the motives which enter into and produce the actions which makes heroe of other men !

When the mist lifted from the valley of the Rappahannock at Fredericksburg on the morning of the 13th December, 1862, a magnificent panorama of battle burst upon the view of the waiting Confederates. Stafford Heights were crowned with Federal guns. Federal troops, in long lines of battle, splendidly equipped and supported by artillery were advancing across the plain, whilst others, in dense masses, were crossing the river on pontoons. Lee's thin line of battle stretched for six miles along the opposite hills of Spottsylvania, awaiting the onset of the mighty hosts of invaders; and the General himself, with Jackson and Longstreet, stood upon an eminence commanding a view of the whole field. It was the grandest field of tourney round which the lists were ever drawn. The Confederate soldier at Fredricksburg that day was impelled to do his duty by motives as strong as ever influenced human action. Love of country, love of home, conviction of right, devotion to principle, knowledge of the great stakes and the great odds, hope and hate, and fear and pride, all nerved his arm; whilst the booming cannon, the flaunting banners, the shrieking shells, the glittering steel, the sharp musketry, the shouts of the advancing foe, the defiant cheers of his comrades, the commands of his officers, and the eyes of his chief, and the All Hail! hereafter which waits upon heroic deeds, fired his heart with the lust of battle and the determination to drive back the invader or to die. He drove the invader back, and the heroic achievment will live forever in story and in song. But he did not exhibit any more nerve than the bold physician who first tied the carotid and external iliac arteries. He did not show more devotion to duty than the assistant surgeon who manfully did his work in the field hospital at Fredericksburg that day. He did not display any more courage than thousands of physicians, every day, who do their duty bravely, knowing that they will never be hailed as heroes and crowned with laurel. But they are heroes in the highest and truest sense of the term.

They enter our houses, are introduced into the penetralia of our homes and received as intimate friends around the hearthstones, on which we offer sacrifices to the Lares and Penates of the household. They are subjected to this severe test of close personal association, in our anxieties, our fears, our humiliations, and our sorrows, and after years of trial we learn to rely upon their strength, their skill, their sympathy and their silence. The coming of such a physician into the home is like the opening of a window to the east, and there are hundreds of households throughout the land in which he is, as Luke was among the apostles, the "beloved physician."

Young gentlemen of the graduating class, in your dreams of professional excellence and success make Him, the physician who represents the class I have thus hurriedly described, your model; and may your dreams be prophets."

